# China Should Increase Fundamental Research on Environmental Health

**DOI:** 10.1289/ehp.12701

**Published:** 2009-05

**Authors:** Aiguo Wang, Xuemin Chen

**Affiliations:** School of Public Health, Tongji Medical College, Huazhong University of Science and Technology, Wuhan City, Hubei Province, China, E-mail: wangaiguo@mails.tjmu.edu.cn

Basic research is the frontier of environmental health. It is the intersection between the charted and uncharted territories of this field, where theoretical and practical breakthrough achievements may take place and where key scientific problems urgently need resolution. Scientific experiments and theoretical work to seek new knowledge, theories, and mechanisms will play a key role in the development of environmental health science. However, fundamental research in China on environmental health is both lacking and late in getting started, in large part because scientific research has historically focused on the study of external environmental factors and the measurement of environmental pollutant concentrations. Not until the mid-1970s, when research gradually combined the environment and health, were there any major scientific studies in China; although this was a step in the right direction for environmental health science, this line of research unfortunately took several years to progress, with little apparent effectiveness. The emergence of this situation has shown that, ultimately, the importance of fundamental research on environmental health has yet to be recognized and has not been given due attention.

There are records documenting the relationship between humans and the environment from ancient times in China. “The Inner Canon of Huangdi” presented a viewpoint of man and earth that is quite similar to the “body–environment interaction” argument of today. This knowledge of ancient China has been practiced and passed down for thousands of years, and still holds theoretical value for guiding or promoting the development of environmental health science today. Such theories, however, are merely a summary of practical experience and are only hypotheses (or assumptions) until they are substantiated by scientific experimentation. The recent rise of molecular biology, the rapid advancement of the Human Genome Project, and the implementation of the U.S. Environmental Genome Project to define “body–environment interactions” has paved the way to important and effective means of research. In particular, the discovery of environmental response genes has provided effective targets for research, and has suggested molecular mechanisms and signaling pathways to explain effects of environmental chemicals. This has not only provided insight regarding body–environment interactions but has also accelerated the research process and launched studies of gene–environment interactions. The development of environmental health science has also been enhanced by new discoveries resulting from the integration of genetic and molecular biology research methods. Therefore, the “harmony between man and nature” theory of ancient China can now be proven by modern scientific experiments.

The wide variety of chemical pollutants in a productive and living environment impacts the health of humans in extremely complicated and harmful ways. Approximately 85,000 synthetic chemicals have entered the marketplace in the past 50 years, with another 1,500 new chemicals being introduced each year. The impact on human health of the vast majority of these products has not been studied. Even the level of toxicity and the mechanisms involved are unknown; therefore, the relationship between these chemicals and human health is far from clear. With the current implementation of the Environmental Genome Project, a new and arduous task has been put forward for environmental health researchers to study environment-related disease pathogenesis. Researchers in the field of environmental health will study such content whether it is relevant to a poisoning mechanism or disease pathogenesis, and the study thereof may help explain why some people in the same environment are poisoned or afflicted with disease while others are not. With the depth of the Environmental Genome Project, this long-standing mystery will gradually be solved; for example, recent research shows that susceptibility to both acute poisonings and chronic diseases may be related to gene polymorphisms that affect the metabolic conversion of environmental pollutants. However, there are many more questions in the field of environmental health science for which we need basic biological research studies in order to reveal its difficulties, questions, and most challenging mysteries.

## Figures and Tables

**Figure:**
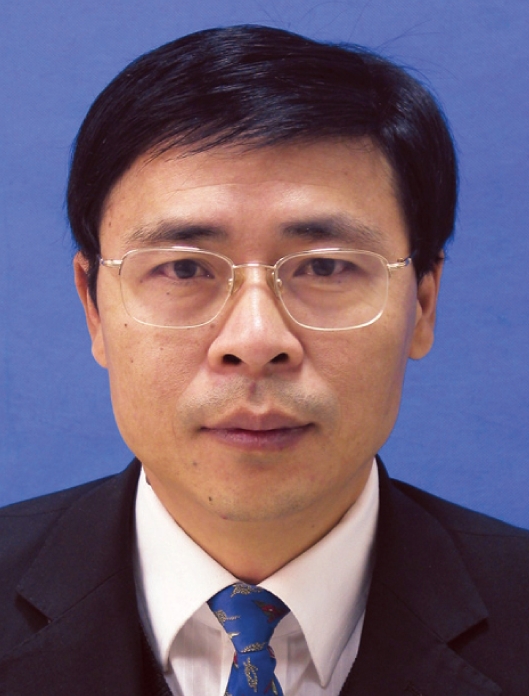
Aiguo Wang

**Figure:**
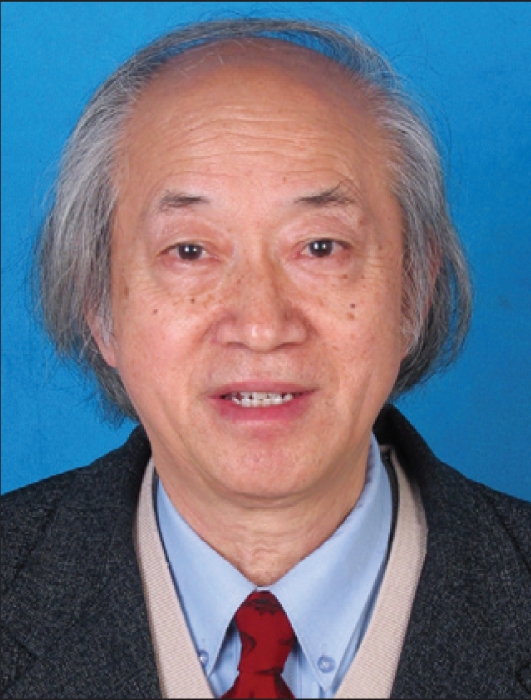
Xuemin Chen

